# The benefits of developing research skills: an ophthalmologist's perspective

**Published:** 2023-01-30

**Authors:** Ciku Mathenge

**Affiliations:** Professor of Ophthalmology: University of Rwanda and Director of Training and Research: Rwanda International Institute of Ophthalmology, Rwanda.

## Abstract

Learning how to carry out research can help you to contribute to innovation in eye care and will expand your scope as a member of the eye team.

Research improves treatments and services for all of society. For ophthalmologists with an interest in research, there are many ways to develop further, such taking an online course for personal or professional development, undergoing training towards a certificate or diploma in clinical or public health research, or even pursuing a research degree such as a master's (MSc) or a doctoral (PhD) degree.

But why would you want to dedicate time and financial resources to this? Won't research become a distraction from your clinical work, and isn't it a waste of your clinical training? These are all valid questions, to be weighed against the many benefits of learning how to carry out research.

## 1. Learning how to carry out research can help you to answer your clinical questions or dilemmas

If you have a passion for discovering or understanding things, the skills you can learn by undertaking a research degree can be useful. Ophthalmologists working in Nakuru Eye unit in Kenya were puzzled when they regularly encountered adult prisoners with xerophthalmia and vitamin A deficiency; conditions that are more often associated with children. One of the ophthalmologists, who was studying for a master's degree, used this opportunity to collect evidence about the relationship between the conditions at the prison and the cases of xerophthalmia they were seeing. This research benefited their patients, and also made their studies more relevant and meaningful.

**Figure:**
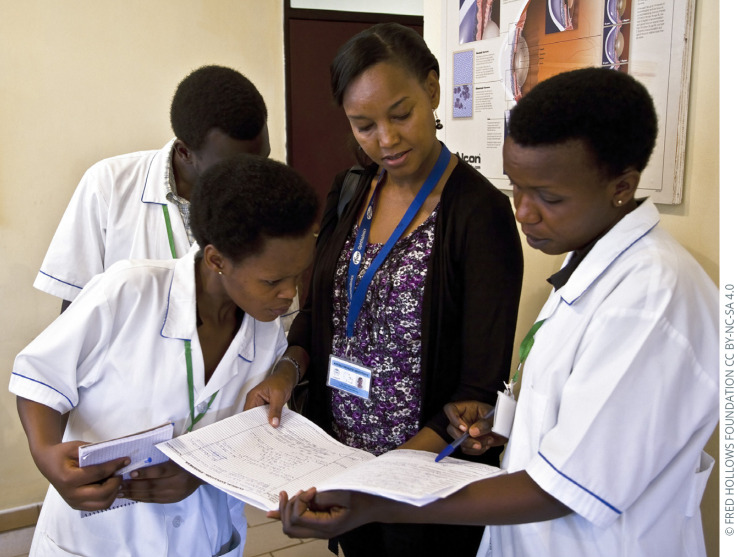
Teaching research skills to other members of the eye team. rwanda

**Figure:**
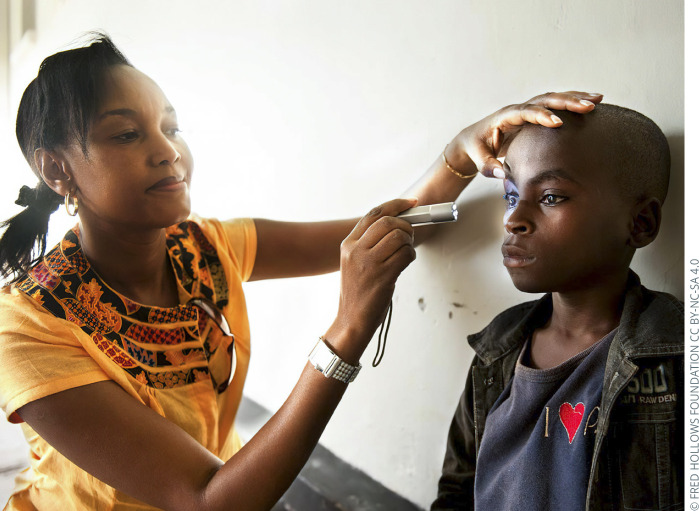
Combining clinical and research work. rwanda

## 2. You will become an expert in your field

A research degree gives you knowledge that you can build on to engage with complex topics in a specialised field. Understanding the epidemiology of retinal diseases motivated one PhD graduate to pursue a retina specialisation. This enabled them to become an expert in the field, and they are now in a position to further our knowledge about retinal ophthalmology and clinical challenges – by both developing theoretical approaches and testing their application through research.

## 3. You will learn how to communicate effectively and in a scientific manner

A research degree, besides helping you to develop expertise in your subject area, also teaches you how to share your findings with your peers and to develop research questions together. You will acquire communication skills that allow you to discuss complex topics with academics, health professionals and peers, both through oral presentations and in writing, including writing and reviewing papers for scientific journals. As you develop your communication skills, actively seeking feedback from others will help you to grow.

## 4. You will become part of a group of engaged peers

During your training, you will learn alongside other motivated individuals, each bringing their own perspectives and experiences to the same problems. Over time, you can develop good working relationships with fellow students and academic staff members, who you can call on and who can call on you for future collaborations. Indeed, some research training institutions reach out to their alumni first when seeking collaborators.

## 5. You will get your academic career rolling

As noted above, a research degree accelerates you towards being a subject matter expert. It is also the most direct pathway to an academic job at a university where a master's or doctoral degree are almost always required. Other opportunities include employment in industrial research and development, or advising on government policy.

## 6. Expanding your network

Being engaged in research is a great chance to expand your network and meet diverse people with similar interests, knowledge, and passion. Taking part in virtual or in-person workshops or seminars, for example, enables you to gain new insights and build connections with other researchers, potential funders, and experts across borders.

## 7. Improves team approach and collaboration in service delivery

Research expands team members’ knowledge about specific problems and broadens everyone's understanding of the roles of each team member. This leads to better understanding of the need for collaborative problem solving and a team approach to service delivery.

In summary, a research degree provides an opportunity for you to undertake extensive and in-depth study in a chosen area of focus. You will gain other skills, for example: skills in analysis and critique, problem-solving skills, improved attention to detail, technological skills (including use of online research tools), and life skills that are fundamental to building a successful career: communication, professionalism, time management, and multitasking.

**Learning research skills can benefit everyone in the eye team**

Research skills can benefit everyone in the eye team. For nurses and clinical officers, developing research skills and/or undertaking a research degree can allow you to support clinical trials, contribute to the growing body of evidence that supports nursing and/or clinical officer practice, and build a career at an academic institution. Many optometrists also build academic and research careers after undertaking a research degree.

Each member of the eye care team has a unique skill set and perspective on eye care practice and delivery. Encouraging everyone with an interest in research to develop those skills and study further makes for very strong research teams that can accomplish great things.

Contributing to research and innovation also supports relevant, evidence-based clinical practice and contributes to education and training for everyone in the eye team; this in turn increases our capacity to reach more patients and provide better eye care.

If you want to see if research is for you, you can try this free online course in ophthalmic epidemiology, which introduces many of the analytical skills that form the basis of a research degree:

https://www.lshtm.ac.uk/study/courses/short-courses/free-online-courses/ophthalmic-epidemiology

As a next step, enquire with universities in your area about what research degree courses are available.

We know of two institutions (one in Cape Town, South Africa and one in London, UK) with master's degrees in public health with a focus on eye care; both have a strong research component. If you know of other institutions that offer similar, high quality courses, please get in touch so we can share their details with other readers of the *Community Eye Health Journal*.

- The University of Cape Town offers the Master's of Public Health (Community Eye Health track) http://www.cehi.uct.ac.za/master-public-health-community-eye-health-track-mphceh
- The London School of Hygiene and Tropical Medicine, which publishes the *Community Eye Health Journal*, offers the MSc Public Health for Eye Care; scholarships are available for eye care professionals working in low- or middle-income countries. See https://www.lshtm.ac.uk/study/courses/masters-degrees/public-health-eye-care

“Encouraging everyone with an interest in research to develop those skills and study further makes for very strong research teams that can accomplish great things.”

**Figure:**
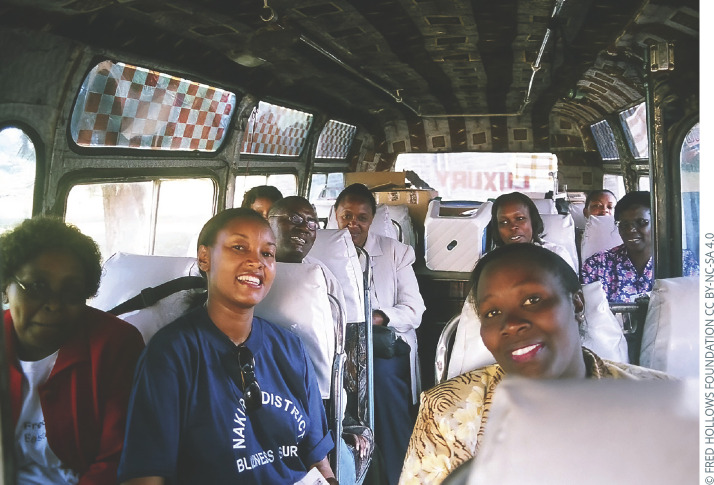
A multidisciplinary research team comprising nurses, ophthalmic nurses, ophthalmic clinical officers, optometrists, and an ophthalmologist on their way to undertake research. kenya

